# Artificial exosomes for translational nanomedicine

**DOI:** 10.1186/s12951-021-00986-2

**Published:** 2021-08-12

**Authors:** Yong-Jiang Li, Jun-Yong Wu, Jihua Liu, Wenjie Xu, Xiaohan Qiu, Si Huang, Xiong-Bin Hu, Da-Xiong Xiang

**Affiliations:** 1 Department of Pharmacy, The Second Xiangya Hospital, Central South University, 139 Middle Renmin Road, Changsha, 410011 China; 2 Hunan Provincial Engineering Research Centre of Translational Medicine and Innovative Drug, Changsha, China; 3 Institute of Clinical Pharmacy, Central South University, Changsha, China

**Keywords:** Artificial, Biomaterials, Drug delivery, Exosomes, Nanomedicine

## Abstract

Exosomes are lipid bilayer membrane vesicles and are emerging as competent nanocarriers for drug delivery. The clinical translation of exosomes faces many challenges such as massive production, standard isolation, drug loading, stability and quality control. In recent years, artificial exosomes are emerging based on nanobiotechnology to overcome the limitations of natural exosomes. Major types of artificial exosomes include ‘nanovesicles (NVs)’, ‘exosome-mimetic (EM)’ and ‘hybrid exosomes (HEs)’, which are obtained by top-down, bottom-up and biohybrid strategies, respectively. Artificial exosomes are powerful alternatives to natural exosomes for drug delivery. Here, we outline recent advances in artificial exosomes through nanobiotechnology and discuss their strengths, limitations and future perspectives. The development of artificial exosomes holds great values for translational nanomedicine.

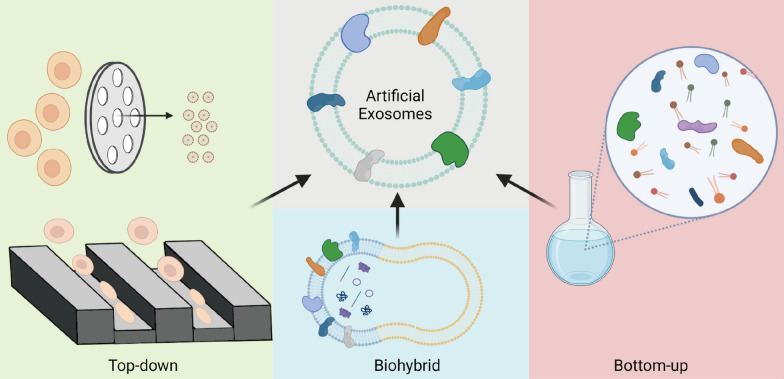

## Introduction

In recent decades, synthetic nanoparticles (NPs) including liposomes [1], micelles [2], dendrimers [3], nanocapsules [4], nanodiamonds [5], nanosponges [6], nanoemulsions [7] and self-assembled peptides [8] have been extensively studied for nanomedicine, particularly for targeted cancer therapy. Drug delivery systems can improve the pharmacokinetic and pharmacodynamics profiles of therapeutics that carried, thereby enhancing the therapeutic effects while reducing undesired toxicity and side effects [9]. Despite undeniable success in the development of nanocarriers for drug delivery in preclinical and clinical studies, the past few decades have witnessed a limited number of approved nanomedicines and most of them are nanoliposomal formulations, Doxil [10–12], Mepact [13], Lipusu [14] and Abraxane [15–18] for example, for anticancer drug delivery.

Exogenous nanomaterials for delivering drugs to the target site may face many hurdles, such as rapid clearance and various biological barriers [19]. It has been reported that only 0.7% of systemically administered NPs can reach the tumor mass [20], despite arguments in a recent reanalysis [21]. Clinical failure of synthetic NPs may be attributed to the differences in the biological barriers and immune systems between human and animal models [22]. NPs could be functionalized by conjugating with polyethylene glycol (PEG) to increase circulation times [23, 24] and by introducing antibodies or peptides [25, 26] to the surface to enhance the targeting capacity and change biodistribution. However, modifications of NPs still can hardly simulate complex biological components. Rapid clearance of PEGylated nanocarriers was observed after repeated administration due to systemic immunogenicity [27]. Besides, a specific ligand modification cannot guarantee high targeting efficiency due to heterogeneity of target cells.

One approach to overcome the limitations of synthetic NPs is developing natural carriers. The field of biological or bioinspired carriers for drug delivery has been advancing. Extracellular vesicles (EVs) [28], which are cell-derived proteolipid membrane vesicles, are emerging in nanomedicine-related fields [29]. Major types of EVs include exosomes, microvesicles, and apoptotic bodies [30]. Our understanding of the between-cell communication has been elevating in the last decade due to EVs, particularly exosomes, which are nano-sized (30–150 nm) subtype of EVs. Exosomes are enriched with various biological components, including proteins, nucleic acids and lipids from their parental cells [31]. Exosome-mediated cell-to-cell communication plays an important role in multiple physiological and pathological processes like tumor metastasis, drug resistance, immune responses and microenvironment homeostasis [32].

Exosomes are also competent candidates for targeted drug delivery [33–35]. Exosomes can escape phagocytosis and achieve long-term circulation by the ‘don’t eat me’ signal for high levels of CD47 on exosomes and trigger CD47-SIRPα interaction that induce immune evasion [36, 37]. In addition, as endogenous cellular compartments, exosomes possess a wide range of cellular adhesion molecules facilitating their penetration through biological barriers. Despite promising results of exosome-mediated drug delivery in murine models, the translation of exosomes is challenged by massive production [38], purification [39], modification [40], drug loading [41] and storage [42]. Also, the heterogeneity between EV subpopulations greatly hindered the quality control for manufacturing and clinical translation [43, 44].

**Fig. 1:**
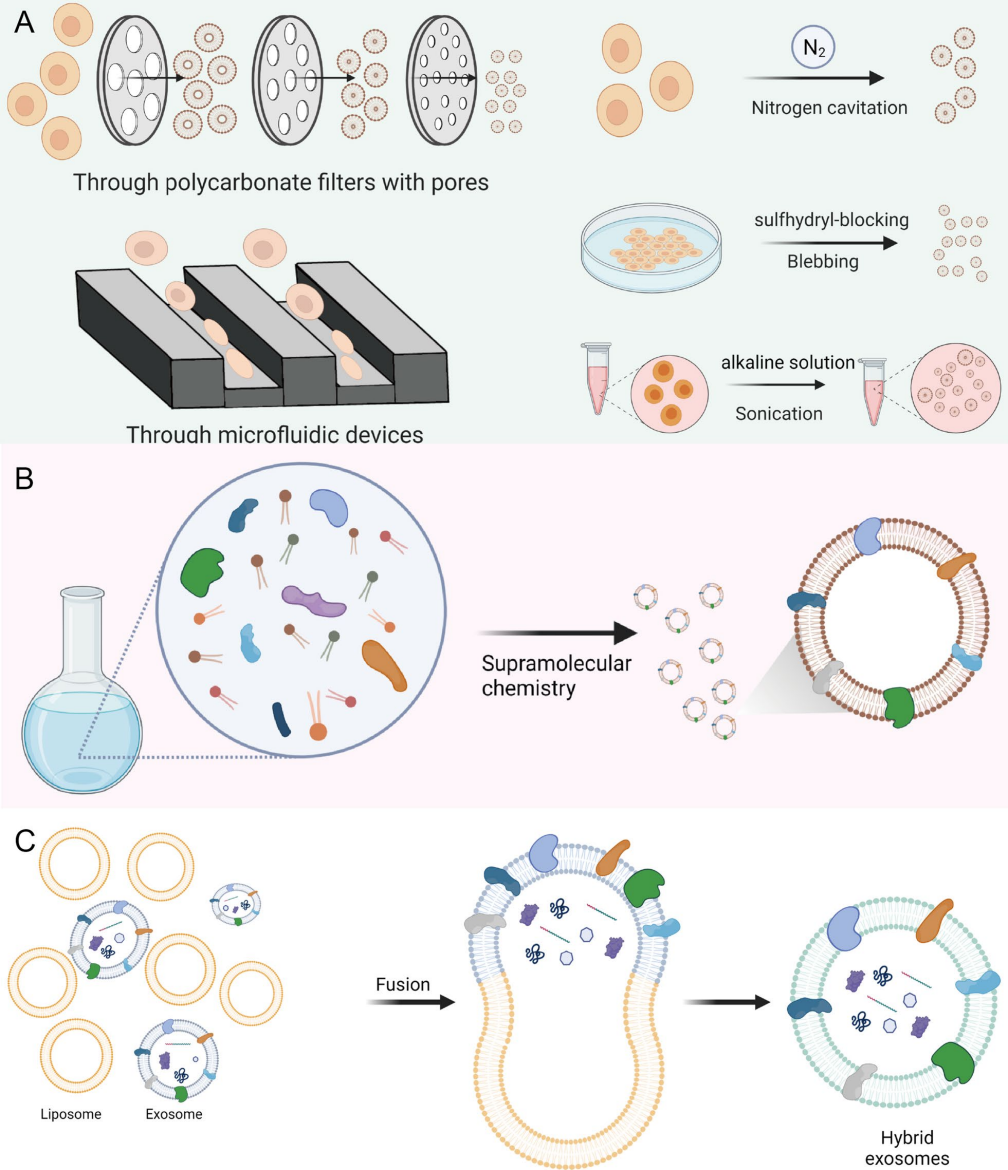
Main strategies for obtaining artificial exosomes based on nanobiotechnology. **A** Top-down strategies for generating nanovesicles (NVs) by manipulating cells. Cells can be forced to pass through membrane pores or microfluidic devices to form NVs; cells can be disrupted by nitrogen cavitation to form NVs; sulfhydryl-blocking can lead to the release of small NVs from cells by cell membrane blebbing; cells exposed to alkaline solution will be broken into membrane sheets, which can form small NVs by sonication. **B** bottom-up strategies for generating fully artificial exosomes by supramolecular chemistry; synthetic materials (lipids) and key components (proteins) from cells can be combined to form exosome-mimetics (EMs) by supramolecular chemistry. **C** Biohybrid strategies for generating hybrid exosomes by fusing exosomes with liposomes. Isolated natural exosomes and synthetic liposome nanoparticles can be fused into hybrid exosomes (HEs) without affecting their intrinsic properties.

In view of the shortcomings of natural exosomes, a growing number of studies are aiming to develop artificial exosomes based on top-down, bottom-up or biohybrid technologies. Those artificial exosomes were generally called ‘nanovesicles (NVs)’, ‘exosome-mimetics (EMs)’ or ‘hybrid exosomes (HEs)’. The development of artificial exosomes through nanobiotechnology hold great promises for advanced drug delivery with combined advantages of natural and synthetic NPs [45]. Here, we provide a comprehensive review of recent advances in nanofabrication of these artificial exosomes (Fig. 1) and discuss their challenges and future perspective for translational nanomedicine.

## Artificial exosomes by nanobiotechnology

### Top-down strategies

For NPs production, top-down strategies have been used by disintegrating big and complex units into elements with smaller sizes. Exosomes are released from cells, herein, the production of artificial exosomes by top-down strategies generally starts from parent cells. Following the top-down strategy, cells are disassembled to form NVs. Therefore, the membrane composition of NVs by top-down strategies is similar to membrane of natural exosomes as they are generated directly from producer cells. NVs from top-down approaches possess natural proteins, nucleic acid or lipids from cells, mimicking the biological complexity of natural exosomes, but may have less heterogeneity. NVs are self-assembled and could be obtained with a few steps [46]. However, the purification methods for NVs are generally based on ultracentrifugation (UC), which is also a time-consuming process commonly used for exosome isolation [47]. Currently, several different fabrication approaches within top-down strategy have been reported for producing NVs by manipulating producer cells (Table 1).

**Table 1:** Summary of artificial exosomes generated by top-down approaches

Cells or materials	Preparation strategy	Particle characterization	Comparison to natural exosomes	Yield	Potential application	Ref.
Human monoblastic U937 cells and mouse macrophage Raw264.7 cells	Serial extrusion (10 μm, 5 μm and 1 μm) + DGUC	Size by NTA: 128 and 133 nm (mode)	Similar morphology, size distribution and protein markers	100-fold	Chemotherapeutic drug delivery	[46]
Mouse fibroblast NIH3T3 cells	Serial extrusion (10 μm, 5 μm and 1 μm) + DGUC	Size by DLS: 150 nm (mean) Zeta potential: − 24.2 ± 0.6 mV	NA	100-fold	siRNA delivery	[48]
Murine embryonic stem cells (D3)	Serial extrusion (10 μm, 5 μm and 1 μm) + DGUC	Size by DLS: about 100 nm	NA	NA	Enhance cell proliferation	[49]
Mouse fibroblast NIH3T3 cells; pancreatic β-cell line MIN6	Serial extrusion (10 μm, 5 μm and 1 μm) + OptiPrep DGUC	3T3-EM: 206.0 ± 19.6 nm; MIN6-EM: 203.8 ± 11.2 nm	NA	100-fold	Induce differentiation of therapeutic insulin-producing cells	[50]
ASCs	Serial extrusion (10 μm, 5 μm and 1 μm) + OptiPrep DGUC	Size by NTA: about 100 nm	Similar size distribution and protein markers	30-fold	Alternative to ASCs for regenerative therapy	[51]
MSCs	Serial extrusion (10 μm, 5 μm and 1 μm) + UC	Size by NTA: about 149 nm	Similar specific protein markers	eightfold	Breast cancer drug delivery	[52]
MSCs	Serial extrusion (10 μm, 5 μm and 1 μm) + DGUC	Size by NTA: about 150 nm	NA	NA	Spinal cord injury treatment	[53]
Human breast epithelial MCF10A cells	Serial extrusion (10 μm, 5 μm and 1 μm) + DGUC	Size by DLS: 131.0 ± 20.5 nm Zeta potential: − 12.6 ± 2.2 mV	Similar size distribution, zeta potential and protein markers	150-fold	siRNA delivery	[54]
Human neuroblastoma SH-SY5Y cells	Serial extrusion (10 μm, 5 μm and 1 μm) + OptiPrep DGUC	Size: 186 nm (mode)	Similar size but distinct proteome	100-fold	Alternative nanocarrier to exosomes	[55]
Human embryonic kidney HEK293 cells	Serial extrusion (10 μm, 5 μm and 1 μm) + DGUC	Size by DLS: 82.15 ± 40.60 nm	Similar specific protein markers	100-fild	RNA delivery for diabetic wounds therapy	[56]
Mice primary hepatocytes	Serial extrusion (10 μm, 5 μm and 1 μm) + OptiPrep DGUC	Size by DLS: 141.1 ± 8.2 nm Zeta potential: − 24 mV	Similar size and zeta potential, and specific proteins	100-fold	Liver tissue repair and regeneration	[57]
Mouse macrophage Raw264.7 cells	Serial extrusion (1 μm, 400 nm and 200 nm) + DGUC	Size by NTA: 189. ± 2.5 nm Zeta potential: − 17.6 ± 0.4 mV	Similar size distribution and zeta potentials	NA	Enhance the efficacy of immune checkpoint inhibitors	[58]
NK92-MI natural killer cells	Serial extrusion (5 μm and 1 μm) + OptiPrep DGUC	Size by NTA: 99.2 ± 21.5 nm	Similar size and morphology, but different protein contents	50-fold	Immunotherapy	[59]
Brain-derived Endothelial bEnd.3 cells	Serial extrusion (5 μm, 1 μm, 400 nm and 200 nm) + UC at 100,000 g	Size by NTA: 141 nm (mode) Zeta potential: − 26.35 ± 0.71 mV	Similar size distribution, zeta potential and protein markers	500-fold	Brain tumor drug delivery	[60]
Mouse macrophage Raw264.7 cells	Nitrogen driven extrusion (100 nm)	Size by NTA: about 120 nm (mean)	Controlled size	NA	NA	[61]
Human breast cancer MDA-MB-231 cells; murine fibroblast 3T3 cells	Magnetic extrusion of IONP-encapsulating endosomes through a nanoporous membrane (200 nm)	231-EM: 151 ± 29 nm 3T3-EM: 162 ± 38 nm	Similar size, morphology and protein composition	tenfold	Tumor drug delivery	[62]
Murine embryonic stem cell line-D3	Centrifugation-based filtration (10 um, 5 um)	Size by DLS: around 100 nm	Similar morphology, size distribution and protein markers	250-fold	Drug and RNA delivery	[63]
Human monoblastic U937 cells	Spin cups (10 μm and 8 μm membrane filters)	Size by DLS: 110 nm (minimal mean) Zeta potential: 2.5 mV	Similar morphology, size distribution, zeta potentials, lipid constituent and protein markers	15-fold	Tumor targeting drug delivery	[64]
Murine embryonic stem cell line-D3	Extrusion through microchannels + OptiPrep DGUC	Size range by DLS: 60–120 nm Zeta potential: − 14.54 ± 1.31 mV	Similar morphology, size distribution and protein markers	NA	Drug and RNA delivery	[65]
Murine embryonic stem cell line-D3	Slicing living cells in microchannels + OptiPrep DGUC	Size range by DLS: 100 to 300 nm	Similar size distribution, protein markers and RNAs	100-fold	Higher encapsulation efficiency for drug delivery	[66]
Human promyelocytic leukemia HL60 cells	Nitrogen cavitation + differential UC	Size by DLS: about 200 nm Zeta potential: − 16 mV	NA	NA	Anti-inflammation therapy	[67]
Human promyelocytic leukemia HL60 cells	Nitrogen cavitation + differential UC	Size by DLS: about 180 nm	Similar size and zeta potential and protein markers	16-fold	Anti-inflammation therapy	[68]
Human monoblastic U937 cells	Sonication + UC	Size by NTA 130 nm (peak)	Similar size distribution and morphology	200-fold	Mitigate systemic inflammatory response by OMVs	[69]
Mouse lymphoma EL4 cells	Sulfhydryl blocking induced by DTT + 30 kDa filtration centrifugation	Size by DLS: 30 nm (mean)	NA	NA	Size control; storage stability; tumor drug delivery	[70]

*ACS* adipose-derived stem cells, *DGUC* density gradient ultracentrifugation, *DLS* dynamic light scattering, *MSCs* mesenchymal stem cells, *NA* not available, *NTA* nanoparticle tracking analysis, *OMVs* Outer membrane vesicles, *siRNA* short interfering RNA, *UC* ultracentrifugation

#### Extrusion-based approaches

Extrusion over polycarbonate membrane filters is widely used to obtain NPs, such as liposomes, with controlled size distribution [71]. By serial extruding through polycarbonate membrane filters with diminishing pore sizes, cells can be turned into NVs with reduced size but maintaining the topology of plasma membrane proteins [46]. For extrusion-based approaches, NVs are generally produced with a simple commercial liposome extruder [72], but other devices have also been designed for scalable and semi-automatic production.

Jang et al. firstly developed bioinspired NVs by extruding human monoblastic U937 cells and mouse macrophage Raw264.7 cells through 10 μm, 5 μm and 1 μm filters, followed by density gradient UC at 100,000 g [46]. They reported that NVs are similar to natural exosomes in terms of morphology, size, protein markers as well as anticancer efficacy after loading of chemotherapeutics. Importantly, NVs have significantly higher yield (100-fold) than natural exosomes. Further, this research group generated NVs from various cells using established protocols and reported different applications including: NVs from embryonic stem cells can enhance cell proliferation [49]; mouse fibroblast NIH3T3 cells derived NVs can be used for efficient siRNA delivery [48]; NIH3T3 fibroblasts and MIN6 pancreatic β-cells-derived NVs can induce in vivo differentiation of therapeutic insulin-producing cells [50]; adipose stem cell (ASC)-derived NVs can serve as alternatives to ACS for similar beneficial effects in animals with emphysema [51]; mesenchymal stem cell (MSC)-derived NVs can be used for breast tumor drug delivery or directly for spinal cord injury treatment [52]. Besides, Yang et al. reported that human breast epithelial MCF10A cells derived NVs-mediated delivery of siRNA to MCF-7 breast cancer cells exhibited significant anticancer efficacy with reduced expression of CDK4 protein [54]; they also found that cellular clathrin-mediated and caveolin-mediated endocytosis play important roles in the uptake pathway of NVs. To understand protein components of NVs compared to exosomes, Kenari et al. generated human neuroblastoma SH-SY5Y cells-derived NVs and natural exosomes and performed the proteomic analysis [55]. They found that NVs and exosomes are similar in size distribution, however, NVs exhibited parental cell-like proteome while exosomes exhibited endosomal-like proteome. Differences in post-translational modification of proteins between NVs and exosomes were highlighted, but this would not affect the potential advantage of NVs as an alternative to exosomes for drug delivery. Tao et al. generated human embryonic kidney (HEK) 293 cell-derived NVs with small size (82.15 ± 40.60 nm) and studied the function of NVs loaded with a high content of LncRNA-H19, which showed a strong ability to neutralize the regeneration-inhibiting effect of hyperglycemia for accelerating chronic wound healing of diabetes mellitus [56]. Obtaining exosomes from primary cells may be more difficult than from cell lines, therefore, generating NVs from primary cells as alternatives to exosomes would be more practicable for translation with high yield and simple production procedures. In this regard, Wu et al. firstly prepared NVs from primary hepatocytes of mice and reported that these NVs are competent to exosomes and can be used as a new option for tissue regeneration medicine [57]. Overall, for the most widely used method, serial extrusion of 10 μm, 5 μm and 1 μm followed by density gradient UC (DGUC), the protocol may be robust, but the yield of NVs compared to natural exosomes may vary significantly from different cell sources (from 8- to 150-fold).

**Fig. 2:**
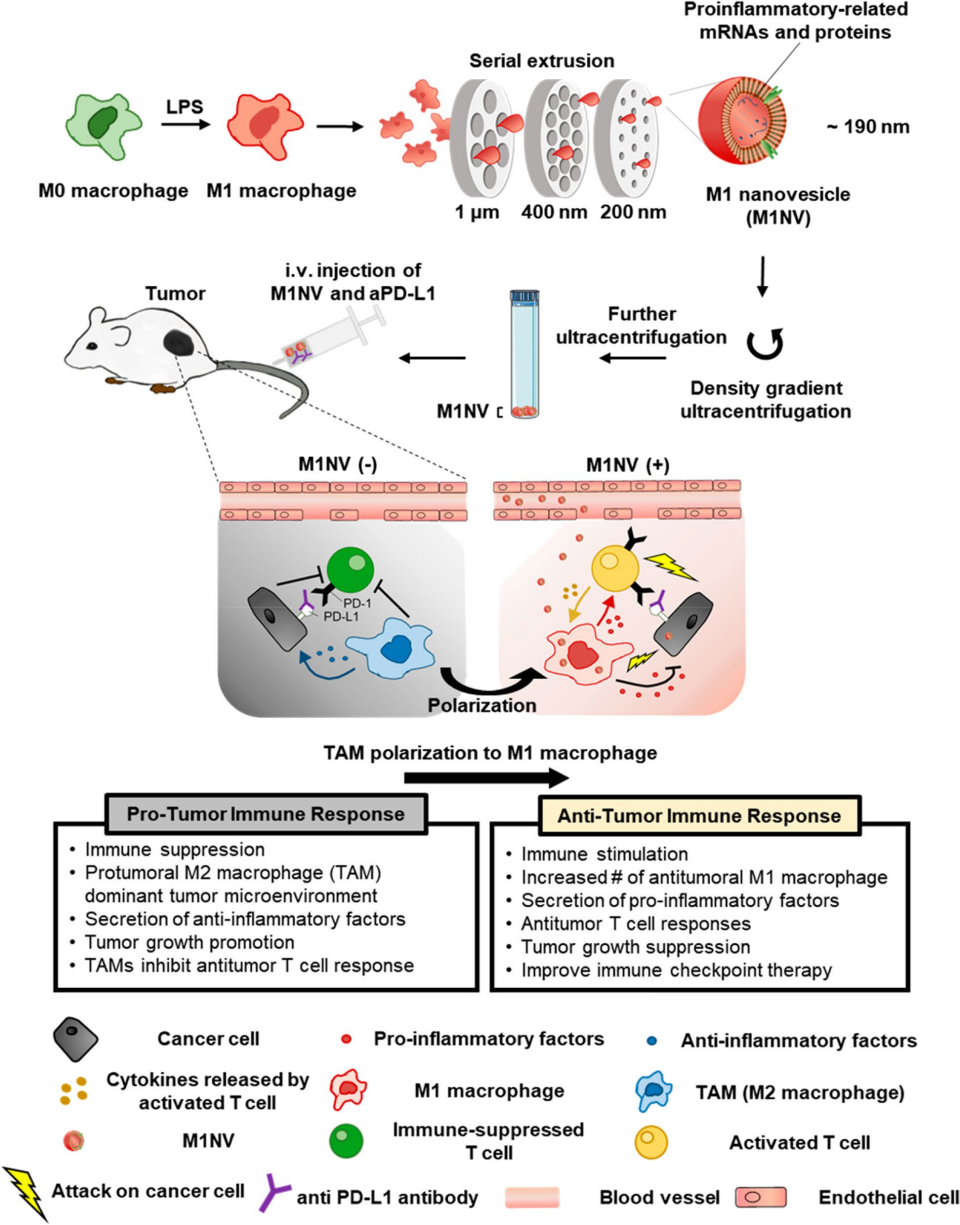
A typical top-down strategy for generating therapeutic macrophage derived-nanovesicles for tumor immunotherapy. M1 nanovesicles (M1NVs) were produced by serial extrusion of M1 macrophage induced by lipopolysaccharide (LPS). M1NV was purified by density gradient ultracentrifugation. M1NV reached the tumor site and polarized M2 tumor-associated macrophage (TAM) to anti-tumoral M1 type and induce secretion of pro-inflammatory cytokines to activate T cells. (Reprint with permission from [58]. Copyright American Chemical Society, 2018)

Steps of extruding and the pore size of filters can be modified for generation of NVs. Choo et al. decreased the pore size of membrane filter to 1 μm, 400 nm and 200 nm and obtained lipopolysaccharide (LPS)-treated RAW264.7 cells-derived NVs as M1NVs (M1 macrophage-derived NVs) [58]. They found that M1NVs could work as an immune regulator to repolarize M2 macrophages in tumors and potentiate the antitumor efficacy of anti-PD-L1. Injection of M1NVs shifted the polarization of microphage in tumors from pro-tumoral M1 type to anti-tumoral M2 type, improving the immune checkpoint therapy (Fig. 2). The substitute potential of NVs to exosomes for immunotherapy was also evaluated in another study with reduced extruding steps (5 μm and 1 μm). Zhu et al. evaluated the cytotoxicity of natural killer cell-derived NVs on cancer cells and reported that these NVs are potent immunotherapeutic agents for the treatment of cancer [59]. Also, extruding steps can be increased depending on the study purpose. We recently generated NVs from brain-derived endothelial cells using serial extruding method with increased steps (10 μm, 5 μm, 1 μm, 400 nm and 200 nm) and reported the highest yield (500-fold) than natural exosomes [60]. Our head-to-head comparison study demonstrated NVs are potent alternative to exosomes for brain tumor nanomedicine.

In addition to serial extrusion using mini extruder, other strategies based on extruding mechanism have also been reported for NVs production. Fan et al. prepared macrophage-derived NVs by one-step nitrogen-driven extruding of living cells through a 100-nm filter [61]. NVs were purified and collected by UC. They show that NVs derived from M1 macrophages have selective tumor-killing effects and the effect can be enhanced by loading chemotherapeutics and constructing a specific miRNA-responded system. However, the yield of NVs by this method was not reported. In another study, Guo et al. enhanced the extruding method for NVs generation by magnetism [62]. They incubated cells with 10 nm iron oxide NPs (IONPs) for endosome encapsulation. Then, IONP-encapsulating endosomes were isolated by hypotonic treatment, homogenization and magnetic separation, followed by nanoporous membrane (200 nm) extrusion. NVs containing IONP were collected by magnetic separation. This method generated relatively pure endosome-derived NVs with very similar size, morphology and protein composition compared to natural small EVs. While the increase in yield is not very high (tenfold), NVs containing IONP can be easily collected without UC. In addition, those endosome-derived NVs can be loaded efficiently with chemotherapeutics by the ammonium sulfate gradient approach.

#### Filtration-based method

The extruding method is effective for NVs production but steps are less controllable as manual operation is required. Similar to the extruding method, the filtration-based method can also be applied to produce NVs using serial membrane filters with different pore size. Protocols of the filtration-based method are more specific than manual extruding and require less manpower.

Jo et al. developed a mechanical device containing membrane filters for large-scale generation of NVs without UC [63]. In the device, cells are forced to pass through membrane pores (10 um and 5 um) by centrifugation. Cells are elongated, ruptured and assembled into NVs by centrifugal force. The NVs obtained by this device showed exosome-like structure and size distribution. While the membrane pores used in the device were relatively large, obtained NVs were small in diameters (~ 100 nm). Also, they reported that NVs generated from murine embryonic stem cells by this method can transfer RNAs to target cells thus activating cellular signaling pathways. Further, Goh et al. simplified the device and reduced the production steps by using spin cups [64]. They obtained U937 cells-derived NVs using spin cups with two membrane filters (10 μm and 8 μm). Also, despite large membrane pores, the size of NVs was slightly larger than nature exosomes. NVs fabricated by the spin cup method showed similarity to exosomes in terms of physical characterization, protein and lipid components. But the yield is not very high (15-fold). Besides, they observed higher targeting effects to tumors of NVs than natural exosomes, demonstrating NVs are ideal and alternative carriers to exosomes for tumor nanomedicine.

#### Microfluidic device-based method

Microfluidic technology is a multidisciplinary and advancing field [73]. Microfluidic devices consist of several micro components for manipulating tiny fluids. Microfluidic systems have many advantages in simplicity, automation and scalable fabrication [74]. Microfluidic systems have shown superiority in the detection, purification, fabrication and engineering of nano-sized materials such as liposomes [75] and exosomes [76].

In recent years, specific microfluidic systems have been developed for the mass production of NVs. Jo et al. used simple pressurization of living cells over a microfluidic device based on an array of parallel hydrophilic microchannels for the production of NVs [65]. Cells are stretched and broken into fragments by the shear stress. NVs are formed by self-assembling. This method efficiently generated NVs with exosome-like characteristics in terms of size, shape and biological contents. Moreover, NVs generated from embryonic stem cells by microfluidic fabrication showed the ability to transfer endogenous mRNA to target cell cultures. The NVs generated in that study exhibited the delivery ability very similar to natural exosomes. Further, this research group designed another microfluidic system for the fabrication of NVs by slicing living cells. Microchannels were lined with the silicon nitride (Si_x_N_y_) microblade (500 nm in thickness) array and polydimethylsiloxane (PDMS) block [66]. Using a syringe pump, cells flowed through the microchannels and microblade that are perpendicularly aligned in the middle of the PDMS channel array, which sliced incoming cells into membrane fragments that were spontaneously self-assembled into NVs. These NVs can be loaded with exogenous materials for efficiently deliver to recipient cells. These two automatic microfluidic device-based methods showed higher yield (100-fold) than natural exosomes; nevertheless, both systems require UC step, which is identical to exosome purification.

#### Nitrogen cavitation-based method

In addition to manipulating living cells via extruding, filtration and microfluidic device, dead cells can also be used to obtain NVs. Gao et al. firstly reported that nitrogen cavitation can efficiently generate EVs-like NVs from neutrophils [67]. Activated neutrophils in suspension were filled in a nitrogen cavitation chamber and were physically disrupted by exposure to nitrogen cavitation at a pressure of 400–500 psi at 0 ℃ for twice. Neutrophil-derived NVs in suspension were isolated by differential UC, followed by sonication. Neutrophil-derived NVs generated by the nitrogen cavitation method retained intact targeting molecules and can selectively bind inflamed vasculature to mitigate acute lung inflammation. Further, they reported that the NVs generated by nitrogen cavitation are similar to natural EVs after systematic comparison of size, morphology and protein markers [68]. However, the increase in yield (16-fold) of NVs by this nitrogen cavitation method is not very high compared to other top-down strategies.

#### Sonication-based method

Fragments of cell membranes may assemble to form small vesicles during sonication. Ghost NVs formed by cell membrane sheets without luminal components are also similar to exosomes. Go et al. broke cells with an alkaline solution and isolated membrane sheets by sonication to discard luminal cytosolic components [69]. Membrane pellets were obtained by UC. Drug-loaded NVs were generated by sonication-induced self-assembling. These NVs have uniformed size and similar physical features compared to natural exosomes but have significantly higher (200-fold) yield. Besides, these NVs can efficiently deliver dexamethasone to endothelial cells to mitigate the systemic inflammatory response induced by gram-negative bacterial outer membrane vesicles.

#### Cell bleb-based method

As for above mentioned top-down strategies, cells are sacrificed by physical force. That means the production of NVs is non-recyclable, limiting the production efficiency. Hence, the generation of natural NVs without sacrificing cells would be an advanced top-down strategy. It would be interesting if mass production of NVs could be achieved without disrupting cells.

A bleb phenomenon has been observed when the cell membrane is exposed to a sulfhydryl-blocking reagent [77]. Despite considerable quantity, sulfhydryl-blocking often induces giant membrane vesicles with high heterologous size distribution that was not suitable for drug delivery. Ingato et al. optimized this sulfhydryl-blocking method by adding paraformaldehyde and dithiothreitol, a disulfide-reducing agent, to cells during culturing [70]. This improved method increased the secretion of natural NVs with a much smaller size (30 nm) after filtration centrifugation. They reported that NVs induced by sulfhydryl-blocking are solely the product of cell membrane blebbing, resulting in high homogeneity. NVs preserved cell membrane components and contain cytoplasmic contents, allowing facile engineering by directly modifying the donor cells. Besides, natural NVs-mediated delivery of doxorubicin (DOX) effectively and safely suppressed tumor growth in a murine tumor model. This strategy showed various advantages in sustainable and massive production, size control, low cost, feasibility and stability.

### Bottom-up strategies

By contrast, bottom-up strategies refer to manufacturing approaches that begin with small molecules as the building block to form large and complex structures through a stepwise assembling process combining their physical and chemical properties. A typical example of the bottom-up strategy in the drug delivery system field is liposome, which is a lipid-based NP designed to mimic the bilayer structure of cell membrane [78, 79]. Also, as natural exosomes share a similar membrane structure to cells, liposomes hold great promise to resemble exosomes [80]. To mimic the structure of natural exosomes, specific lipids, from classical formulations to simulating the composition of exosomes, are used to form lipid bilayer and then modified with chemical groups or engineered with surface proteins. Therefore, bottom-up strategies require a deep understanding of the functions of key components of natural exosomes. By assembling desired components learned from natural exosomes, artificial exosomes can be clean in composition and have controllable characteristics. Therefore, from an industrial view, fully artificial exosomes may have higher pharmaceutical acceptability and would be more suitable for production and in concordance with regulation. In addition, one of the most widely used method for liposome preparation is the thin layer evaporation (TLE) method. Lipids are firstly dissolved in organic solutions. By evaporating the solution, lipids could form a thin layer in the flask. Lipid suspension can be obtained by adding water (hydration) to the lipid thin layer. Extruding the lipid suspension through membrane pores will produce liposomes with controlled size distribution. During the TLE process, drugs in a solution can be incorporated into liposomes during hydration, leading to a more efficient drug loading for artificial exosomes than pre or post-loading drugs into NVs or exosomes [81]. Several bottom-up approaches to fabricate artificial exosomes start from synthetic materials have been summarized (Table 2).

**Table 2:** Summary of artificial exosomes generated by bottom-up approaches

Preparation	Particle characterization	Comparison to natural exosomes	Advances	Application	Refs.
Liposomes (PC, CHOL, DSPE-PEG_2000_, DSPE-PEG-MAL) coupled with MHC peptide complexes	Size by TEM: about 100 nm	NA	Targeted and traceable artificial exosomes	Activate and expand functional antigen-specific T cells	[82]
Liposomes (PC, SM, Chol, DOGS-NTA) bind with APO2L, TRAIL-His10	Size ranged by DLS: 150 –200 nm	NA	Artificial exosomes with improved bioactivity	Effective treatment of antigen-induced arthritis	[83]
Liposomes (PC, SM, Chol, DOGS-NTA-Ni) with inserted recombinant Apo2L, TRAIL	Size ranged by DLS: 150 –200 nm	NA	Artificial exosomes with improved bioactivity	Overcoming Chemoresistance of Human Hematologic Tumor Cells	[84]
Nanoliposome (Cremophor EL, PC, DOPE, DC-Chol) coupled with DEC205 monoclonal antibody	Size by DLS: 81.64 ± 4.25 nm Zeta potential: 19.8 ± 1.8 mv	NA	Targeted artificial exosomes High encapsulation efficiency	Specifically transmit antigen to DCs to induce immune responses	[85]
Liposomes (DOPC, SM, Chol, DOPS, DOPE) coated chitosan with embedded Cx43 proteins	Size by DLS: 120 ± 4 nm Zeta potential: -8 ± 2 mv	Similar size and specific protein marker	High biocompatibility Effective cytosolic delivery capability	Biomimetic siRNA delivery vehicles	[86]
Liposomes (Chol, PC, SM, Cer) tailored with integrin α6β4	Size by DLS: 113 ± 1 Zeta potential: − 5 ± 2	Similar size, structure and delivery efficiency	Production methodology and regulations	Targeted delivery of therapeutic oligonucleotides to lung cancer	[87]
Liposomes (DMPC, DSPC, DOPC, Chol) and leukocyte membrane proteins	Size by DLS: 122 ± 1.2 nm Zeta potential: − 14 mv	NA	Large-scale and fast preparation Stable structure High drug-loading capacity Custom-tailored functionality	Targeted tumor therapy	[88]
Liposomes (DMPC, DSPC, DOPC, Chol) and tumor cell membrane proteins	Size by DLS: 115 nm (mean) Zeta potential: − 45.6 ± 1.0 mV	NA	Large-scale and fast preparation Multifunction Stability	Targeted tumor penetration	[89]
Liposomes (DPPC, DSPC, DOPC, Cholesterol) and hybrid cell membrane proteins (tumor cell and red blood cell)	Size by DLS: about 100 nm Zeta potential: − 17 mv	Similar size and protein markers	Large-scale and fast preparation Stable structure High drug-loading capacity Multifunction	Anti-phagocytosis capability and targeted tumor therapy	[90]
TPE-BPA, CTAB and Fe ions	Size by DLS: less than 100 nm	NA	Fundamental understanding of natural fission–fusion processes of exosomes	Molecular configuration and siRNA delivery	[91]

*Cer* Ceramide, *Chol* Cholesterol, *CTAB* cetyltrimethylammonium bromide, *DC* Dendritic cells, *DC-Chol* 3-(*N*-(*N*0,*N*0-dimethylaminoethane)carbamoyl)Cholesterol, *DLS* dynamic light scattering, *DOGS-NTA* 1,2-dioleoyl-*sn*-glycero-3-{[N-(5-amino-1-carboxypentyl)-iminodiacetic acid]succinyl}(nickel salt), *DOPC* 1,2-dioleoyl-*sn*-glycero-3-phosphoCholinev *DOPE* 1,2-dioleoyl-*sn*-glycero-3-phosphoethanolamine, *DOPS* 1, 2-dioleoyl-*sn*-glycero-3-phosphoserine, *DPPC* 1,2-dipalmitoyl-*sn*-glycero-3-phosphocholine, *DSPC* 1,2-distearoyl-*sn*-glycero-3-phosphocholine, *DMPC* 1,2-dipalmitoyl-*sn*-glycero-3-phosphoCholine, *DSPE-PEG* 1,2-distearoyl-*sn*-glycero-3-phosphoethanolamine-*N*–[methoxy(polyethylene glycol)2000] , *DSPE-PEG-MAL* 1,2-distearoyl-*sn*-glycero-3-phosphoethanolamine-*N*-[maleimide (polyethylene glycol)-2000], *MHC* Major histocompatibility complex, *NA* Not available, *PC* Phosphatidylcholine, *siRNA* short interfering RNA, *SM* sphingomyelin, *TEM* transmission electron microscope; TRAIL-His10

#### Liposomes conjugated with specific peptide

Conjugating liposomes with peptide is a simple way to develop artificial exosomes; however, which peptide to be conjugated is dependent on what kind of bioactivity is supposed to have in artificial exosomes. Classical liposomes coupled with synthetic MHC Class I/peptide complexes as artificial exosomes could mimic functions of exosomes [82]. Specifically, MHC/peptide complexes and Fab regions were incubated with Traut’s reagent for binding to maleimide lipids. After removing excess Traut's reagent by using a desalting column, activated MHC/peptide complexes and Fab regions were incubated with liposomes, followed by chromatography and ultrafiltration for purification. These artificial exosomes showed similar size to natural exosomes and effectively targeted to T cells and worked semi-directly to activate and expand T cells. Similarly, Martinez-Lostao et al. generated liposomes with a size and lipid composition resembling natural exosomes [83]. APO2L/TRAIL was conjugated with liposomes. The resulting artificial exosomes showed considerable therapeutic effects on antigen-induced arthritis in rabbits. Similarly, in another study, the same formulation of liposomes was used and conjugated with Apo2L/TRAIL with improved activity [84]. These artificial exosomes successfully overcame the chemo-resistance of human hematologic tumor cells. For liposomes conjugated with peptides, the key might be the activity of the peptide, while liposomes worked mostly as a carrier.

#### Liposomes coupled with specific antibody

Similar to conjugating liposome with peptide, liposome coupled with antibody may also exert bioactivity mostly for the specific antibody. To mimic the function of exosomes and target dendritic cells (DCs) to activate immune, Li et al. utilized DC-Chol, 3-(N-(N0, N0-dimethylaminoethane)carbamoyl)Cholesterol, to provide positive charge and developed cationic nanoliposomes by micro-emulsion and micelle assembling [85]. DEC205 monoclonal antibody was anchored to nanoliposomes via reactions of amine group of DEC205 and ester, activated by adding 1-(3-Dimethylaminopropyl)-3-ethylcarbodiimide hydrochloride (EDC) and *N*-Hydro xysuc cinimid (NHS), of the nanoliposomes. This strategy provided an efficient approach to prepare artificial biomimetic exosomes to develop antigen carriers for specific DCs targeting and antigen presentation to induce immune responses.

#### NPs embedded with specific protein

In addition to mimic the functions of exosomes to activate molecular pathways, liposome-based artificial exosomes can also be used for drug or gene delivery. To develop EM NPs for siRNA delivery, Lu et al. constructed liposomes coated chitosan NPs as exosome-mimicking membranes to protect siRNA cargo and introduced to synthesized plasmids encoding connexin 43 (Cx43), a transmembrane protein, to direct the transcription, translation, and integration of Cx43 in the lipid layers in EM [86]. Integrated Cx43 worked functionally in cellular transport and facilitated the delivery of siRNA in EM to Cx43-expressing U87 MG cells. The EM exhibited high siRNA delivery efficiency and biocompatibility. Despite limited delivery efficiency than commercial transfection Lipo 2000 reagent, that strategy formulated EM using a cell-free protein synthetic approach and advanced the development of artificial exosomes as biomimetic nanocarriers.

In another study, Vázquez‑Ríos et al. developed EM nanosystems that simulate exosomal structure and functions for targeted drug delivery to lung adenocarcinoma cells [87]. Liposomes were loaded with therapeutic oligonucleotides and then tailored with integrin α6β4 for lung organotropism. The EM showed great similarities to natural exosomes from physicochemical and pharmaceutical aspects. The EM exhibited similar lung targeting and delivery efficiency to tumor-derived exosomes. Importantly, this strategy provided important advantages in terms of production methodology and regulations. Still, for liposomes modified with a specific protein, the protein is vital for simulating the functions of natural exosomes.

#### Liposomes modified with membrane proteins

Modification of specific proteins on liposomes may endow desired functions such as enhanced cellular uptake and targeting effects, but it may be inadequate to resemble the complex functions of natural exosomes. Proteomic study revealed thousands of functional proteins in exosomes [55], indicating impossibility to perform engineering modification repeatedly. A feasible approach would be integrating a group of proteins with one step.

It has been reported that leukocytes freely circulate in the bloodstream and selectively target the inflamed vasculature in response to injury, infection, and tumorigenesis [92]. Leukocyte-derived NVs can evade the mononuclear phagocytic system and are able to across the endothelial vessel [93]. Molinaro et al. extracted membrane proteins from circulating leukocytes in the blood and developed leukocyte-mimicking biomimetic liposomes, called leukosomes, by incorporating membrane proteins in lipid vesicles [88]. Membrane proteins from leukocyte possess cellular adhesion molecule ligation, such as lymphocyte function-associated antigen 1, macrophage-1 antigen, and P-selectin glycoprotein ligand-1, etc., facilitating the delivery of DOX to both tumor-associated vasculature and parenchyma, which might be difficult for specific protein modification. Chemotherapeutic-loaded leukosomes could be used for targeted tumor therapy with potent anti-cancer activity. That study demonstrated the versatility of liposomes modified with membrane proteins as artificial exosomes for biomedical applications. Recently, we also generated biomimetic liposomes by incorporating cancer cell membrane proteins into synthetic liposomes and enhanced the tumor targeting ability [89]. Further, elastase was bound to biomimetic liposome through charge-mediated interaction. Biomimetic liposomes with surface bound elastase degraded tumor stroma and facilitated tumor penetration of chemotherapeutics and cytotoxic T lymphocytes.

**Fig. 3:**
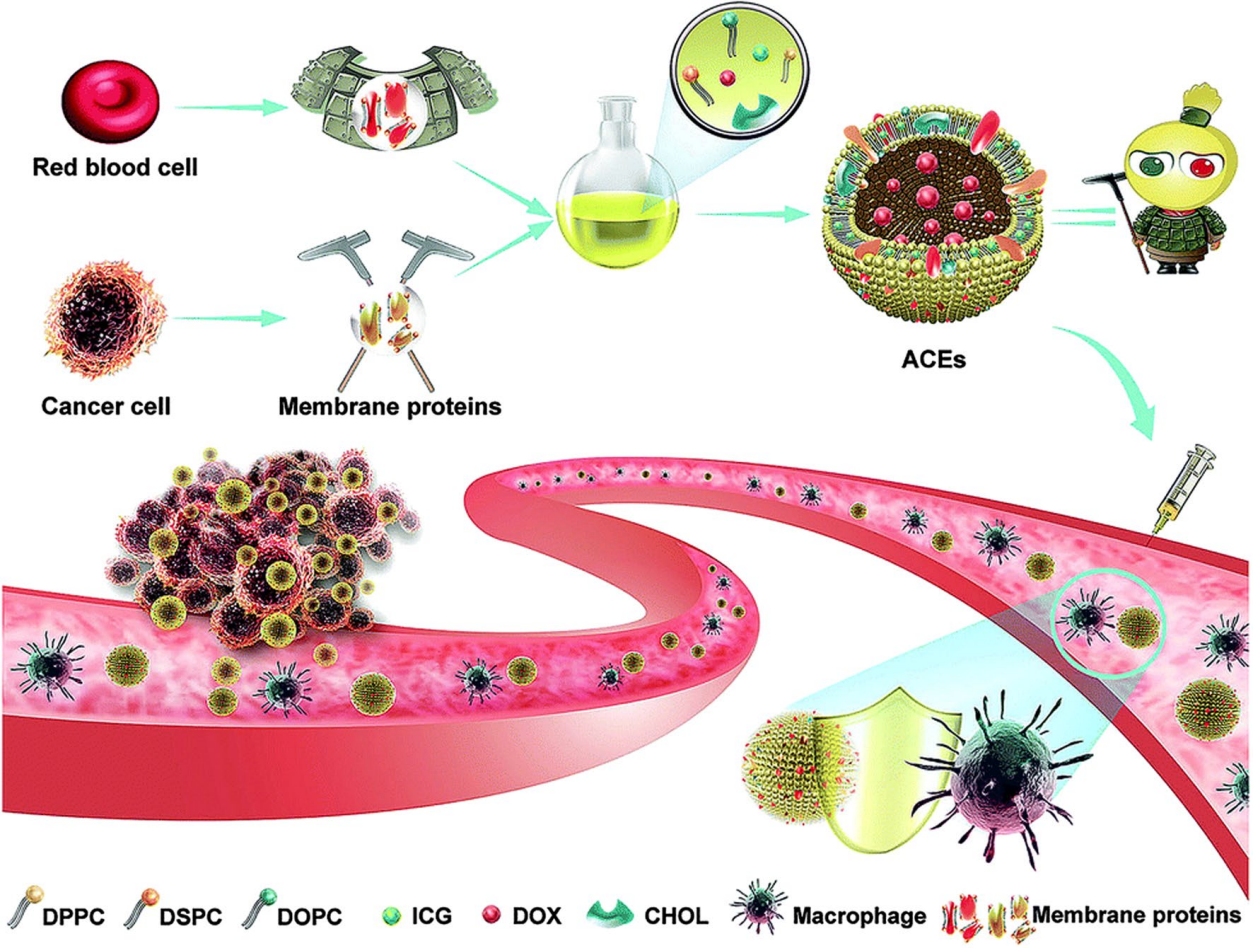
A typical bottom-up strategy for generating fully artificial exosomes for drug delivery. Hybrid membrane proteins from red blood cells and cancer cells were integrated into liposomes during preparation to form artificial chimeric exosomes (ACEs). ACEs have anti-phagocytosis ability during circulation (from red blood cell) and the tumor-homing ability (from cancer cell) for targeted drug delivery. (Reprinted with permission from Ref. [90]Copyright Royal Society of Chemistry, 2019)

In addition to membrane proteins from specific cell types, membrane proteins from different cells could also be simultaneously used to modified liposomes, depending on desired functions, as hybrid artificial exosomes. Zhang et al. used hybrid membrane proteins strategy and developed protein chimeric liposomes as artificial chimeric exosomes (ACEs), combining anti-phagocytosis properties from red blood cell (RBC) membrane proteins and tumor targeting abilities from homologous tumor cell membrane proteins [90]. More specifically, the anti-phagocytosis capability was from the high level of CD47 in membrane proteins of RBCs while tumor targeting and adhesion abilities were from EpCAM, Galectin 3 and N Cadherin in membrane proteins of cancer cells (Fig. 3). That strategy demonstrated that hybrid membrane proteins are useful for developing multifunctional artificial exosomes.

#### Fully synthetic EM without proteins

The investigation of the fission (releasing) and fusion (swallowing) processes of exosomes are important for the understanding of intercellular transport and signaling. However, manipulating natural exosomes has been challenging due to natural complexity in composition and cellular environment. In this regard, Li et al. developed supramolecular vesicles consisted of aggregation-induced emission active molecule TPE-BPA and cetyltrimethylammonium bromide (CTAB) [91]. The TPE-BPA@8CTAB vesicles are capable of coordination with metal ions such as Fe^2+^ and Fe^3+^. While the vesicles have no protein component, they can fuse together and form large-sized vesicles upon oxidation, undergo a fission process and then return to small-sized vesicles through reduction, mimicking the fusion and fission behaviors of exosomes. The aggregation-induced emission (AIE) feature from TPE-BPA of the EM enabled monitoring of vesicular transformation by fluorescence emission changes [94], thus providing innovative understanding for the fusion and fission behaviors of exosomes, especially for cargo release. Besides, the EM may be a candidate for drug delivery as it can release siRNA through the fusion process. While this supramolecular vesicle may not be competitive for drug delivery compared to other artificial exosomes for the lack of protein components, this type of EM may serve as a useful tool for innovative understanding of exosome behaviors.

### Biohybrid strategies

In addition to top-down and bottom-up strategies, biohybrid is another technique that has been used for developing EMs by merging synthetic NPs and natural vesicles. Biohybrid is an emerging field and these hybrid vesicles are designed specifically for application in advanced drug delivery with optimized performance. EM by biohybrid strategies is semi-artificial with the advantages of synthetic materials including production, controlling, engineering, drug loading and stability and properties of endogenous exosomes such as tropism, biological barrier penetration, long circulation, low immunogenicity and high biocompatibility. A number of studies have developed hybrid of nanomaterials and natural vesicles, mostly by fusion exosomes with liposomes, while the fusion process may be achieved by different techniques (Table 3).

**Table 3:** Summary of artificial exosomes generated by biohybrid approaches

Synthetic nanomaterials	Natural vesicles	Biohybrid approach	Particle Characterization	Comparison to natural exosomes	Advances	Application	Refs.
Liposomes (DOPC, DOTAP, DSPE-PEG_2000_)	Raw264.7 cell-derived exosomes	Freeze-thawing	Size by NTA: 190 – 230 (average)	Increased size and similar protein markers	Membrane surface engineering	Exosome modification	[95]
Liposomes (DPPC, DSPE-PEG_2000_, MSPC	Genetically engineered fibroblast-derived exosomes	Freeze-thawing	Size by NTA: 135.7 nm (average) Zeta potential: − 8.2 mV	Similar morphology and protein markers	Lipid engineering of exosomes	Thermo-sensitive chemoimmunotherapy	[96]
Liposomes (lipofectamine 2000)	HEK293FT cell-derived exosomes	Direct incubation	Size distribution by DLS: 50 –1000 nm	Increased size, but similar protein markers	Efficient encapsulation of large plasmids	CRISPR/Cas9 system transfer to MSCs	[97]
Liposomes (POPC, DOPE)	HUVEC-derived EVs	Incubation with PEG-mediated fusion	Size distribution by NTA: 50 –400 nm	Increased size but similar morphology and protein markers	Efficient EV cargo loading and delivery	Drug loading and delivery	[98]
Liposomes (L-a-phosphatidylcholine and cholesterol)	Mouse macrophage J774A.1 cell-derived sEVs	Extruding (400 and 200 nm)	Size by DLS: 177 ± 21 nm Zeta potential: − 26 ± 3 mV	Increased size but similar protein markers	Colloidal stability Drug loading and pH-sensitive sustained drug release	Tumor targeted drug delivery	[99]
Lipids (DOTAP, POPC, DPPC or POPG)	EVs derived from fibroblast 3T3 cells or A549 lung cancer cells	Extruding (400, 200 and 100 nm)	Size by DLS: around 100 nm	Similar size and with native EV fractions	Mass production (6- to 43-fold vesicles)	Efficient siRNA delivery	[100]
Liposomes (cholesterol, DOPC, DSPE-PEG_2000_)	Murine fibroblast L-929 cell-derived exosome	Extruding (400 and 200 nm)	Size by DLS: about 125 nm Zeta potential: − 7.1 mV	Similar size and protein markers	Efficient cargo loading targeting	Pulmonary anti-fibrotic drug delivery	[101]

*DLS* Dynamic light scattering, *DOPC* 1,2-dioleoyl-*sn*-glycero-3-phosphocholine, *DOPE* 1,2-dioleoyl-*sn*-glycero-3-phosphoethanolamine, *DOPS* 1,2-dioleoyl-*sn*-glycero-3-phospho-l-serine, *DOTAP* 1,2-dioleoyl-3-trimethylammonium propane, *DPPC* 1,2-dipalmitoyl-*sn*-glycero-3-phosphocholine, *DSPE-PEG*_*2000*_ 1,2-distearoyl-sn-glycero-3-phosphoethanolamine-*N*-[methoxy(polyethylene glycol)-2000], *EV* Extracellular vesicles, *HUVEC*Human Umbilical Vein Endothelial Cell, *MSC* Mesenchymal stem cell, *MSPC* 1-stearoyl-2-hydroxy-*sn*-glycero-3-phosphocholine, *NTA* Nanoparticle tracking analysis, *POPC* 1-Palmitoyl-2-oleoyl-*sn*-glycero-3-phosphocholine, *POPG* 1-Palmitoyl-2-oleoyl-*sn*-glycero-3-phosphoglycerol, *siRNA* short interfering RNA

#### Biohybrid by freeze-thawing

Despite tropism of exosomes, the membrane engineering may be necessary for advanced drug delivery [102]. To increase colloidal stability and blood circulation of exosomes by optimizing the surface, Sato et al. applied a facile membrane-engineering strategy using direct membrane fusion between synthetic liposomes and purified exosomes [95]. To induce the fusion of exosome and liposome membranes the authors used a freeze–thaw method previously developed for liposome engineering. Exosome–liposome mixture was subjected to liquid nitrogen and thawed at room temperature for several cycles. The mean size was increased and protein components were diluted after fusion. The exosomes-liposomes hybrid, as advanced drug delivery systems, showed increased cellular uptake than liposomes and can transport exogenous hydrophobic lipids from liposomes and hydrophilic cargoes from exosomes to recipient cells.

**Fig. 4:**
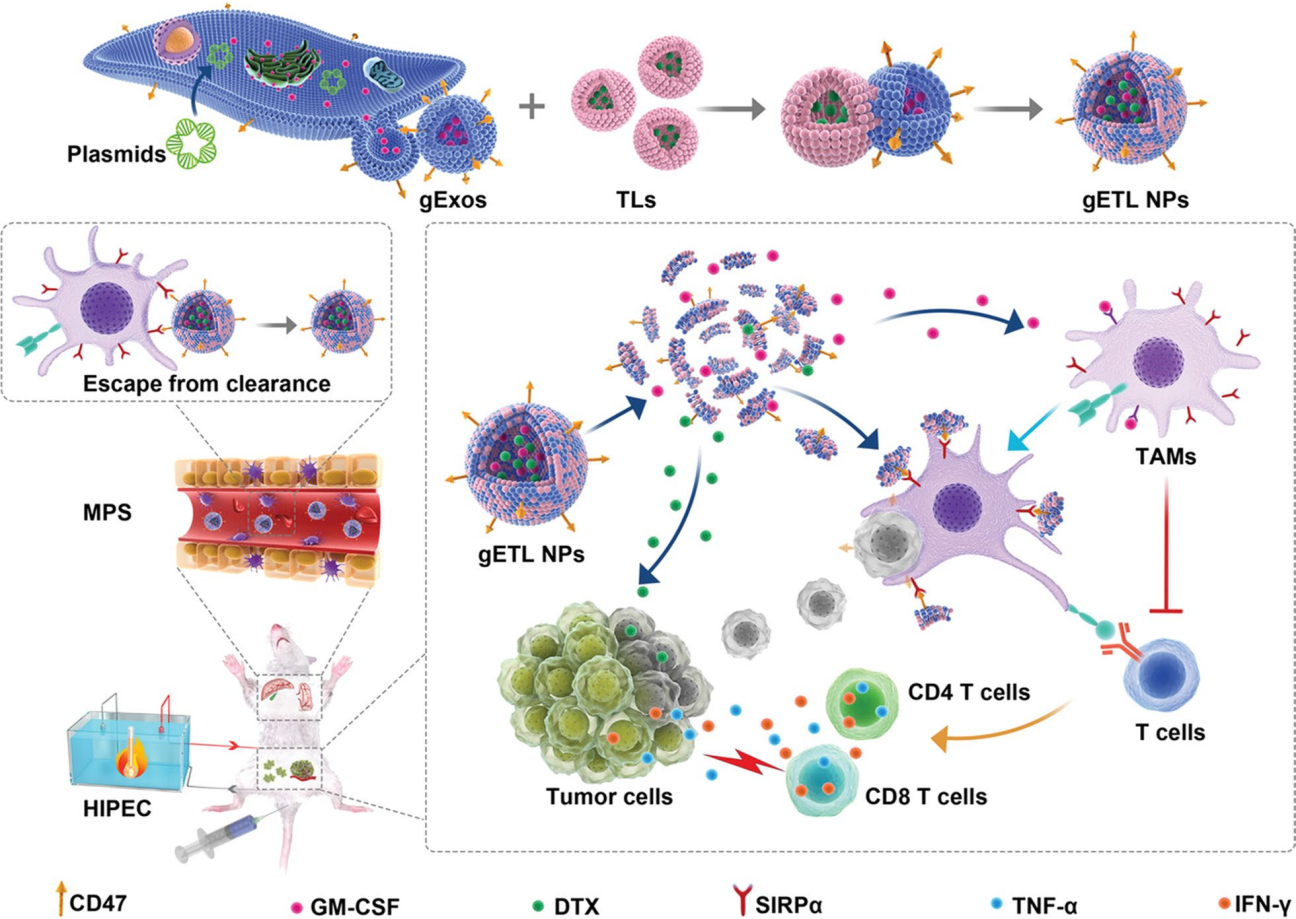
A typical biohybrid strategy for generating hybrid exosomes by freeze-thawing induced fusion for combined tumor chemo-immunotherapy. Genetically engineered exosomes (gExos) were fused with thermosensitive liposomes (TLs) to form hybrid nanoparticles (gETL NPs), which can escape from clearance of mononuclear phagocytic system (MPS) and be activated by hyperthermic intraperitoneal chemotherapy (HIPEC) at the tumor site to release drugs for chemotherapy and polarize tumor-associated macrophages to M2 type to activate T cells for tumor immunotherapy. (Reprinted with permission from Ref [96]. Copyright WILEY–VCH 2020)

Synthetic liposomes can be multifunctional such as pH-sensitive [103], photo-sensitive [104] and thermosensitive [105] by incorporating specific lipids liposomes. Fusion multifunctional liposomes with natural vesicles would be a feasible approach for endowing exosomes with enhanced delivery properties and developing multifunctional hybrid. Lv et al. generated hybrid NPs of genetically engineered fibroblasts-derived exosomes expressing CD47 and thermosensitive liposomes following freeze–thaw procedures [96]. The hybrid NPs are slightly large in size but have similar morphology to exosomes and retained most protein markers of exosomes. Importantly, the hybrid NPs showed improved circulation and preferential accumulation in tumors and released drugs in response to temperatures. Genetically engineered thermosensitive liposome-exosome hybrid can escape mononuclear phagocytic system and target the tumor site, in which hyperthermia therapy stimulates the hybrid and induces combined immune-chemotherapy (Fig. 4).

#### Biohybrid by incubation

Incubation is a mild and commonly used method for various cellular processes and reactions. Fusion of liposomes and exosomes may occur during incubation as their membranes both have lipid bilayer structure. Incubation-induced spontaneous fusion may result in wide size distribution of hybrid particles with a high polydispersity index (PDI). The impact of incubation conditions on the fusion process requires further investigation.

Exosomes are promising targeted delivery nanocarrier, but have limited efficiency, for their small size, in encapsulating exogenous large nucleic acids, such as CRISPR/Cas9 System. The fusion of exosomes and liposomes may achieve efficient loading of large plasmids into the hybrid. Lin et al. developed a hybrid NP by simple incubation of HEK293FT cell-derived exosomes with CRISPR/Cas9 expressing liposomes at 37℃ for 12 h [97]. The hybrid NPs delivered the CRISPR/Cas9 system to mesenchymal stem cells (MSCs) and achieved gene editing by expressing the encapsulated genes in the MSCs, which cannot be transfected by the liposome alone. Taken together, the exosome–liposome hybrid NPs can deliver CRISPR–Cas9 system in MSCs, providing a promising tool for targeted gene editing.

The incubation-induced fusion of exosomes and liposomes can be enhanced by mediators on the surface, such as PEG. Piffoux et al. modified the EVs by incubation with liposomes via PEG-mediated fusion [98]. PEG-mediated fusion was proved to be an efficient approach to engineer EVs with exogenous compounds while preserving their inherent contents. Moreover, this fusion method enabled bioengineering of liposomal particles with biogenic molecules; importantly, the PEG-mediated fusion strategy allows efficient loading of cargoes and the feasible engineering of EV membranes with adaptable functions.

#### Biohybrid by co-extruding

Another reported method for generating biohybrid of exosomes and liposomes is co-extrusion. Under physical stress, membranes of exosomes and liposomes would break and re-assemble to form hybrid vesicles when passing through the membrane pore with controlled size. Exosomes have been explored as a drug delivery candidate owing to their natural functionalities. However, it has been reported that exosomes obtained by different method may differ in yield and purity [47]. Unlike freeze-thawing and incubation biohybrid methods, which are spontaneous, the co-extruding method has advantages in controlling the product hybrid vesicles.

Liposomes have pharmaceutical flexibility for engineering and modification for preparing functional carriers but lack endogenous functionality compared to natural vesicles. Rayamajhi et al. hypothesized that macrophage-derived sEVs possess tumor-targeting properties for targeted drug delivery [99]. Macrophage J774A.1 cell-derived sEVs (139 nm) and synthetic liposomes (131 nm) at the ratio of 1:5 were mixed, vortexed, sonicated and extruded through polycarbonate membrane filters (400 and 200 nm). The hybrid vesicles showed a slightly larger size (177 nm) than precursors but have a more uniformed size distribution (PDI: 0.19) than sEVs (PDI: 0.25). Advantages of the two types of nanocarriers were merged as efficient hybrid delivery systems.

The low yield of EV by natural secretion limits its mass production and potential for clinical applications. Engineering EVs by introducing exogenous lipids can tune their surface composition and functionality and increase their production without affecting intrinsic targeting and delivery properties. For membrane engineering of EVs, the synthetic lipid can be directly used for co-extruding with EVs without liposome formation. Jhan et al. mixed EVs with suspension of synthetic lipids and serially extruded through membranes (400, 200 and 100 nm) to form lamellar vesicles with controlled size [100]. This method increased the number of vesicles by 6 to 43-fold than isolated EVs. Also, exogenous siRNA was successfully loaded into hybrid vesicles that retained improved cellular uptake to recipient cells and achieved an effective gene silencing effect comparable to commercial Lipofectamine RNAiMax. Doping synthetic lipids in membranes of natural EVs is a convenient approach for maximizing the delivery potentials with increased particle quantities.

More recently, the delivery potentials of hybrid of exosomes and liposomes were assessed in vivo for targeted pulmonary fibrosis therapy, which has been challenging in the clinic for insufficient drug concentration and poor targeting. Sun et al. developed a clodronate (CLD)-loaded hybrid drug delivery system, consisted of liposome and fibroblast-derived exosomes with properties preventing phagocytosis and targeting fibroblast, for the treatment of pulmonary fibrosis [101]. The HEs efficiently accumulated in the fibrotic lesion and exhibited significant penetration of pulmonary fibrotic tissue for the improved affinity for fibroblasts by homologous exosome.

## Comparison of natural exosomes and artificial exosomes

Along with the rapid growth in nanobiotechnology, the research of exosomes has been advancing from biology [32, 106] to biomarkers [107, 108] and nanomedicines [109] over the past decade. For drug delivery, exosomes have shown various advantages compared with conventional synthetic materials such as liposomes [110]. The therapeutic potential of exosomes-mediated drug delivery are still in tests of preliminary clinical trials (pancreatic cancer: NCT03608631; acute ischemic stroke: NCT03384433; colon cancer: NCT01294072), while the efficacy of cell-derived exosome-like vesicles has been evidenced in several pilot trials [111–113]. Nevertheless, clinical translation of natural exosomes has been challenging [114]. Major hurdles including large-scale production, standard purification protocols, characterization of complex composition, cargo loading, quality control and storage stability are in their way to products for therapeutic applications [115]. Mass production of exosomes may be achieved through the development of bioreactors [38]; however, as biological components, their standardized and reproducible production requires comprehensive control of genetic stability and culturing condition of producing cells; purification requires subtype identification and quantification of contaminants [116]; efficacy is dependent on drug loading efficiency and capacity [117]; storage of therapeutic exosomes is supposed to have high recovery without damage to exosome particles as well as their biological contents [118, 119]. From a current perspective, the development of exosomes for therapeutic drug delivery is still in infancy and the cost for translational research and clinical application would be very high.

In recent years, artificial exosomes have been developed with higher pharmaceutical acceptability to overcome the drawbacks of natural exosomes as new theranostic biomaterials for potential clinical applications [45]. However, there are some challenges for different strategies developing artificial exosomes for the following aspects: yield, procedures, time–cost, manpower, sustainability, characterization and efficacy, which are summarized and compared to natural exosomes (Table 4).

**Table 4:** Comparison of natural exosomes and different types of artificial exosomes for translational nanomedicine

Types	Source	Scalability	Procedures	Time-cost	Manpower	Sustainability	Characterization	Applicability
Natural exosomes	Cell supernatant	★^a^	☆☆^b^	☆☆☆	☆☆☆	★★	★	★★
Artificial exosomes (top-down approach)	Cells	★★	☆☆	☆☆	☆☆	★	★	★★★
Artificial exosomes (bottom-up approach)	Synthetic materials	★★★	☆☆	☆	☆	★★★	★★	★
Artificial exosomes (biohybrid approach)	Synthetic materials and cell supernatant	★	☆☆☆	☆☆☆	☆☆☆	★★	★	★★★

^a^★ is to indicate that the aspect is favourable

^b^☆ is to indicate that the aspect is unfavorable

Top-down strategy is the most widely reported method for obtaining artificial exosomes. One major strength of the top-down strategy is the applicability because NVs are fully biological and have similar physiochemical and biological features to natural exosomes. Similar to natural exosomes, the yield of artificial exosomes by top-down strategies may vary from cell to cell. For the serial extruding method, most studies reported a nearly 100-fold higher yield than natural exosomes, but higher yields have also been reported and the maximum was 500-fold (Table 1). Compared to natural exosomes, preparation of artificial exosomes by top-down strategies could be cumbersome as UC-based purification procedures are still required following serial extruding or nitrogen cavitation. The development of specific devices may simplify the procedures and increase production efficiency and reduce time–cost and manpower [63]. However, for generating NVs, cells are sacrificed and broken into fragments, leading to limited production sustainability. Also, it has not been raised whether natural exosomes in cells should be considered as contaminants that may influence the characterization of NVs.

Bottom-up strategies are able to produce “clean” artificial exosomes with determined formulations and have the highest scalability because synthetic materials could be feasibly obtained and used for massive production. Besides, the cost of time and manpower could be remarkably reduced by using synthetic materials. Multiple modifications of liposomes are still challenging for complex procedures, uncertain conditions and instability. Another major drawback of bottom-up approaches for generating liposome-based artificial exosomes is that synthetic materials can still hardly mimic the complex composition of natural exosomes. Therefore, the functions of natural exosomes can hardly be fully reproduced by artificial exosomes based on bottom-up strategies. Publications that have compared artificial exosomes with natural exosomes are scarce. Most studies only evaluated physicochemical and biological properties of artificial exosomes (Table 2). A previous study reported a preliminary comparison concerning drug delivery efficiency [87]. Currently, preparing artificial exosomes that fully assembles components of natural exosomes may be pharmaceutically impossible [64], modification of liposomes with key proteins is dependent on the purpose and may not be consistent with natural exosomes. Therefore, the translational applicability of bottom-up strategies is limited.

Biohybrid approaches can only produce semi-artificial exosomes. The yield of artificial exosomes by biohybrid strategies would not be very high as natural exosomes are still required. In addition, the involvement of natural vesicles may face methodological challenges similar to natural exosomes. Preparation of biohybrid exosomes could be laborious as preparation of synthetic liposomes and isolation of natural vesicles are both required. Characterization of hybrid particles may be influenced by additional liposomes and exosomes and the purification would be rather difficult as liposomes, exosomes and the hybrid are very similar in multiple aspects. However, one strength of the biohybrid approach is that the hybrid possesses natural components from exosomes, despite dilution, those hybrid nanocarriers may have improved delivery efficiency than liposomes and higher stability than exosomes, leading to high applicability. Besides, the fusion method that is widely used in biohybrid strategies provided a feasible option for drug loading such as loading biological cargoes into liposomes and loading exogenous therapeutic agents into exosomes [98].

## Concluding remarks

Biomimetic nanocarriers are the next generation of drug delivery systems in nanomedicine for improving health. Advancements in nanobiotechnology provide avenues for the development of artificial exosomes that may accelerate clinical translation for nanomedicine application. Currently, natural exosomes are just in their preliminary clinical trials and artificial exosomes are not yet ready for translation. Major challenges include the preparation protocols, characterization and biocompatibility concerns. Artificial exosomes have commercial advantages for their up-scale productivity. In the future, novel and multifunctional artificial exosomes will be developed, with contributions from multidiscipline efforts of biotechnology, nanotechnology, chemical engineering and pharmaceutical industry, to improve healthcare. We hold confidence for artificial exosomes’ potentials for personalized nanomedicine.
